# Metabolic plasticity in CLL: adaptation to the hypoxic niche

**DOI:** 10.1038/leu.2015.187

**Published:** 2015-09-04

**Authors:** K M Koczula, C Ludwig, R Hayden, L Cronin, G Pratt, H Parry, D Tennant, M Drayson, C M Bunce, F L Khanim, U L Günther

**Affiliations:** 1 School of Cancer Sciences, University of Birmingham, Birmingham, UK; 2 School of Biosciences, University of Birmingham, Birmingham, UK; 3 Department of Haematology, Birmingham Heartlands Hospital, Birmingham, UK; 4 School of Immunity and Infection, University of Birmingham, Birmingham, UK

## Abstract

Metabolic transformation in cancer is increasingly well understood. However, little is known about the metabolic responses of cancer cells that permit their survival in different microenvironments. We have used a nuclear magnetic resonance based approach to monitor metabolism in living primary chronic lymphoid leukemia (CLL) cells and to interrogate their real-time metabolic responses to hypoxia. Our studies demonstrate considerable metabolic plasticity in CLL cells. Despite being in oxygenated blood, circulating CLL cells are primed for hypoxia as measured by constitutively low level hypoxia-inducible factor (HIF-1α) activity and modest lactate production from glycolysis. Upon entry to hypoxia we observed rapid upregulation of metabolic rates. CLL cells that had adapted to hypoxia returned to the ‘primed' state when re-oxygenated and again showed the same adaptive response upon secondary exposure to hypoxia. We also observed HIF-1α independent differential utilization of pyruvate in oxygenated and hypoxic conditions. When oxygenated, CLL cells released pyruvate, but in hypoxia imported pyruvate to protect against hypoxia-associated oxidative stress. Finally, we identified a marked association of slower resting glucose and glutamine consumption, and lower alanine and lactate production with Binet A0 stage samples indicating that CLL may be divided into tumors with higher and lower metabolic states that reflect disease stage.

## Introduction

Chronic lymphocytic leukemia (CLL) is the most common form of leukemia in Western countries^1, 2, 3^ which despite recent improvements in prolonging survival, remains incurable.^1, 4^ CLL patients present with elevated lymphocyte counts in the peripheral blood. In most patients these lymphocyte numbers increase progressively over months and years. However, these circulating cancer cells are out of cell cycle and superficially highly quiescent. Despite this, isotopic labeling studies have determined that peripheral blood CLL cells have undergone a number of divisions and also that rates of cell death within the tumor are high.^5^ The picture that emerges is that circulating CLL cells represent a large pool of non-dividing cancer cells that are able to enter and exit tissue sites, predominantly lymph nodes, spleen and bone marrow, wherein they proliferate and drive the progressive expansion of the tumor.^5^ Entry into tissue sites provides important survival signals that protect against chemotherapeutics and thus lead to relapsed disease.^6^ Therefore, understanding how peripheral blood CLL cells can survive transitions between normoxic and hypoxic conditions is likely to identify novel strategies to tackle this disease. Furthermore, studying CLL as an unusual cancer where a large proportion of cells within the tumor exhibit motility between different sites in the body may permit the potential discovery of mechanisms pertinent to metastasis of other cancers.

Studies of cancer cell metabolism have enjoyed a recent renaissance with the recognition that altered cancer cell metabolism is a critical component of the tumor phenotype that can provide opportunities for biomarker discovery and the derivation of novel therapeutic approaches.^7, 8, 9, 10^ The renewed interest in cancer metabolism has been fueled by the advent of metabolomics technologies, in particular, the ability to perform complex simultaneous and non-targeted analyses of multiple metabolites using either nuclear magnetic resonance (NMR) or mass spectrometry (MS) platforms. To date, the majority of studies have focused on the interrogation of cell extracts. Performing time-course analyses in this way requires sufficient biological material to permit multiple extractions. Although possible with cell lines, this is a limiting factor for studies using primary human cells. Studies based on extracts are also less able to study the dynamics of cell plasticity to changing environmental factors and their interaction with their metabolic environment. Here we present an NMR based technology that allows the real-time study of metabolism in primary patient blood-derived CLL cells. To our knowledge, this is the first report of real-time NMR measurements using non-modified or cultured primary patient cancer cells. We have used NMR to study real-time metabolism in CLL cells in response to changing oxygenation levels. Using one-dimensional ^1^H-NMR spectra we achieve a time resolution of 5–8 min. We observe that ‘quiescent' CLL cells appear to be primed for hypoxia and display remarkable plasticity of metabolic adaptation that is associated with hypoxia-inducible factor-1 (HIF-1α) activity and which displays functional changes in the protective utilization of pyruvate. Our data also suggest that it may be possible to identify that CLL cell metabolism differs with disease stage.

## Materials and methods

### Primary CLL cells

Patients with B-cell CLL attending the outpatient clinic at Birmingham Heartlands Hospital and Queen Elizabeth Hospital were randomly selected for this study. The patients had been diagnosed according to standard morphologic, immunophenotypic and clinical criteria (Oscier, Dearden *et al.* 2012) and samples were obtained following informed consent and ethical committee approval (10/H1206/58).

Our studies used a total of 76 samples provided by 63 individual patients. Sixty of the 76 samples were from 50 patients who had never undertaken treatment for their CLL. A further two samples came from patients believed to be untreated but for whom the records were incomplete. Ten samples came from patients who had historical treatment but were untreated for 17 months to 14 years before sampling. Only four samples were from patients on active management; one had three courses of bendamustine in the 6 months before sampling; one had received R-CHOP within 12 months of sampling, one was having intermittent chlorambucil and the fourth patient intermittent chlorambucil/fludarabine.

Mononuclear cells were isolated from venous blood using Leucosep tubes (Greiner Bio-one, Gloucester, UK) loaded with 15-ml Ficoll-Paque plus (G.E Healthcare, Amersham, UK) according to manufacturer's instructions. CLL cells were not further purified to avoid their activation. All but three mononuclear cell samples contained >80% CLL cells as determined by CD19 positivity. The non-CD19 positive cells in each sample where not characterized. Mononuclear cells were cultured at 5 × 10^6^ cells/ml in RPMI 1640 (Invitrogen Gibco, Paisley, UK) supplemented with 1% ITS+ (BD Biosciences, Oxford, UK), 100 U/ml penicillin and 100 μg/ml streptomycin (Invitrogen Gibco) in a humidified incubator at 37 °C and 5% CO_2_. Cell viability was assessed using annexin V and propidium iodide co-staining using an AV-FITC kit (BD), according to manufacturer's instructions. Stained samples were analyzed on a BD FACSCalibur (BD) using the BD CellQuest software.

### Real-time NMR sample preparation

CLL samples with >80% Annexin V negativity were selected for NMR experiments. Cells were suspended at 5 × 10^7^ cells/ml or 1 × 10^7^ cells/ml in 1 ml 0.1% w/v low melting agarose (Sigma-Aldrich, Dorset, UK) in serum free, bicarbonate buffered RPMI 1640 medium, in a CO_2_-free atmosphere, supplemented with 1% v/v ITS+ (BD Biosciences) and 1 mM sodium 3-(trimethylsilyl)propionate-2,2,3,3-d4 (Cambridge Isotope Laboratories, Tewksbury, MA, USA) as an NMR chemical shift standard and 10% D_2_O (GOSS Scientific Instruments Ltd., Crewe, UK) for lock stabilization. A volume of 600 μl of cell suspension was loaded into NMR tubes and an oxygen sensor connected to a Fiber Optic Oxygen Meter (World Precision Instruments, Hitchin, UK) inserted through the hole in the NMR cap before sealing.

### Real-time NMR measurements

One-dimensional NOESY spectra were acquired at 37 °C, using a 500 MHz Bruker spectrometer (Bruker, Coventry, UK) equipped with a cryogenically cooled probe. The spectral width of the acquired spectra was 12 p.p.m., with 32,768 acquired complex data points. The transmitter frequency offset was 4.696 p.p.m. and the water resonance was suppressed by presaturation. For apodization, an exponential multiplication window function with a line broadening of 0.3 Hz was used and the NMR data was zero filled to 32,768 points. Measurements were carried out with deuterium frequency locking after shimming. For time course experiments, a series of 144 one-dimensional spectra were acquired over 24 h. Each spectrum was obtained within ~10 min of sample injection to the magnet acquiring 64 transients.

### NMR time-course data analysis

NMR data was processed using NMRLab^11, 12^ in the MATLAB (The Mathworks, Natick, MA, USA) programming environment. During data processing, spline baseline was applied to all of the 170 spectra using the MetaboLab software.^12^ All spectra were aligned to the 3-(trimethylsilyl)propionate-2,2,3,3-d4 signal. NMR resonances of metabolites were assigned and concentration of pyruvate calculated using the Chenomx software (http://www.chenomx.com) and HMBD (Human Metabolite Database, http://www.hmdb.ca). Kinetic modeling was carried out using MATLAB (Mathworks, Cambridge, UK).

### NMR pH measurement

The difference between the chemical shifts corresponding to imidazole ring protons H2 and H5 (attached to C2 and C5) of histidine was used to calculate the pH value as previously described.^13^ To calculate the pH for each NMR spectrum a calibration curve for the H2 and H5 histidine protons was determined in a solution of RPMI medium adjusted to pH 3–8.5 (Supplementary Figures S4A and B). The pH value was calculated using the equation: pH= 549+log10 ((δ−1272)/(0.7004−δ)).

### Flow cytometry

Reactive oxygen species assay: carboxy-H_2_DCFDA (Molecular Probes, Invitrogen, UK) was dissolved in dimethyl sulfoxide (DMSO) to yield a 10 mM stock and stored under nitrogen at −20 °C. Carboxy-H_2_DCFDA was added to 500 μl CLL cell suspension to a final concentration of 10 μM and incubated at 37 °C for 45 min. After incubation, cells were analyzed by flow cytometry (emission wavelength 517–527 nM) (Becton Dickinson FACSCalibur using Becton Dickinson CellQuest software). Mitochondrial superoxide assay: MitoSOX Red (Molecular Probes, Paisley, UK) was used to assess the presence of mitochondrial superoxide. Before use, MitoSOX Red was freshly dissolved in DMSO to yield a 5 mM stock and subsequently diluted to a working concentration of 5 μM in warm phosphate-buffered saline. CLL cells were centrifuged, washed with warm phosphate-buffered saline and resuspended in 200 μl of staining solution before incubation at 37 °C for 10 min. Mitochondrial superoxide accumulation was analyzed by flow cytometry (emission wavelength 580 nm) (Becton Dickinson FACSCalibur using Becton Dickinson CellQuest software).

### Western blotting

Cells were lysed (5 × 10^6^) CLL in RIPA buffer and 30 μg proteins separated by SDS–polyacrylamide gel electrophoresis. Proteins were transferred to Immobilon-P membrane (Millipore Corp, Bedford, MA, USA) and probed with primary antibody overnight at 4 °C (anti-HIF-1α—BD Biosciences, anti-GLUT1- Santa Cruz Biotechnology (Dallas, TX, USA), anti-VEGF- Abcam (Cambridge, UK), anti-LDHA-Abcam, anti-β-actin-Sigma-Aldrich) followed by secondary anti rabbit (Sigma-Aldrich) or anti mouse conjugated with horseradish peroxidase (Sigma-Aldrich). Signal was developed using Supersignal West Pico Chemiluminescent substrate (Pierce, Northumberland, UK) and detected by exposure to Kodak Xomat imaging film (Sigma-Aldrich). Films were developed using an AGFA CURIX 60 (Agfa, Mortsel, Belgium).

### RNA isolation and quantitative real time PCR

Total RNA was isolated using the RNeasy kit as per manufacturer's instructions (Qiagen, Manchester, UK). SuperScript II Reverse Transcriptase (Invitrogen, Paisley, UK) and random hexamers were used for synthesis of complementary DNA. Quantitative real time PCR reactions were performed using an ABI Prism 7700 sequence detector (Applied Biosystems, Paisley, UK) using SensiFast SYBR Hi-Rox kit (Bioline, London, UK) and gene-specific Quantitect primers (Qiagen): VEGF (Hs_VEGFA_1_SG), GLUT1 (Hs_SLC2A1_1_SG) and LDHA (Hs_LDHA_1_SG). Three biological replicates were used for each of the target genes, with each sample assessed in triplicate. Results were normalized to the internal reference gene 18S rRNA.

### Treatments

Chetomin (CTM) (Sigma-Aldrich) was dissolved in DMSO and used at concentrations 10–100 nm. Cells were pre-treated with CTM in media at 37 °C for 3 h before transferring into hypoxic conditions for a further 21 h. Alpha-cyano-4-hydroxycinnamate (Sigma-Aldrich) was dissolved in DMSO and used at concentrations 2–5 mM. Cells were pre-treated for 3 h before transferring into hypoxic conditions. Sodium pyruvate (Sigma-Aldrich) was prepared in deionized water and used at a final concentration of 5 mM.

### Statistical analysis

Student's *t*-test was used for statistical analyzes where indicated.

## Results

### Real-time metabolism monitored by NMR

**Figure 1:**
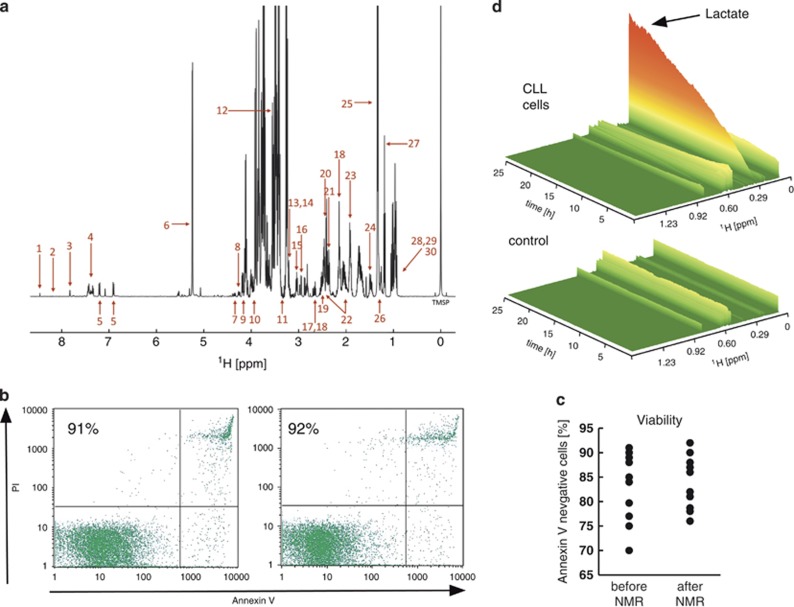
CLL cells survive NMR analyzes and display marked metabolic activity. Primary-CLL mononuclear cells were monitored for 24 h in the NMR at 37°C. (**a**) Representative one-dimensional-^1^H NMR spectrum of CLL cells in 0.1% agarose in serum-free bicarbonate buffered RPMI 1640 growth medium. Metabolites assigned: 1-formate, 2-hypoxanthine*, 3-histidine, 4-phenylalanine, 5-tyrosine, 6-glucose, 7-trans-4-hydroxyl-L-proline, 8-uridine, 9-pyroglutamate, 10-serine, 11-myo-inositol, 12-glycine, 13-phosphocholine, 14-choline, 15-lysine, 16-asparagine, 17-aspartate, 18-methionine, 19-glutamine, 20-succinate, 21-pyruvate, 22-glutamate, 23-arginine, 24-alanine, 25-lactate, 26-3-hydroxybutyrate, 27-ethanol, 28-valine, 29-isoleucine and 30-leucine. *hypoxanthine was detectable after a few hours of the time course. Data shown is representative of >25 primary CLL samples. Viability of CLL cells was assessed pre- and post-NMR analysis by (**b**) monitoring cell morphology of Jenner–Giemsa stained cell cytopsins, and (**c**) Annexin V/propidium iodide (PI) staining and flow cytometry. Viable cells are identifiable as Annexin V/PI negative (lower left quadrant). Percentage viable cells is indicated in the scatter plot. Data shown is representative of >25 samples. (**c**) Viability data for 10 primary CLL samples. (**d**) Representative three-dimensional view of an NMR time course experiment. The control sample contained RPMI medium with ITS+ and 0.1% low melting point agarose (no cells), whereas the second panel contained additionally 5 × 10^7^ CLL cells/ml. Metabolite intensity is highlighted by a color gradient and height. The tallest visible orange peak corresponds to lactate. Data shown are representative of three CLL samples.

NMR experiments were conducted in 5-mm NMR tubes using 5–10 × 10^7^ primary CLL cells suspended in 0.1% agarose in serum-free RPMI growth medium to prevent their sedimentation during the acquisition of spectra over 24 h. We obtained line widths of 1–1.5 Hz in 0.1% agarose (Figure 1a) suggesting that this matrix preserves the mobility of small molecules, probably arising from large cavities in the polymer. Using ^1^H-NMR spectra we obtained sufficient sensitivity to obtain one-dimensional spectra in 5–10 min in a standard 5-mm NMR tube from which ~35 metabolites could be identified (Supplementary Figure S1).

Importantly, the cells recovered from the NMR tube after 24 h displayed no significant changes in viability or morphology (Figures 1b and c). The ability to recover viable cells during the time course of each experiment not only verified the validity of our approach but also meant that cells could be used for subsequent downstream analyzes such as western blotting and quantitative real time PCR.

### Out of cycle CLL cells show high metabolic activity

Primary peripheral blood CLL cells are out of cell cycle (Supplementary Figure S2) and have a relatively scant cytoplasm (Figure 1b), features commonly associated with quiescence. Despite this, we observed marked metabolic activity in these cells over 24 h, evidenced most notably by growth in signals for lactate (Figure 1d). Control spectra recorded for media and agarose without cells confirmed that the observed changes in intensities arose from metabolic activity of the CLL cells (Figure 1d). The volume of 0.6–3 × 10^7^ primary CLL cells is <5 μl in the NMR tube (~0.5–1% total sample volume) suggesting that NMR detects predominantly extracellular metabolites. This was confirmed by comparing the final one-dimensional-^1^H-NMR spectra recorded from CLL cells in media+agarose with the spectra acquired from media alone after cells and agarose had been removed by centrifugation. The spectra obtained were almost identical (Supplementary Figure S3).

Other studies have focused on extracellular acidification rate that is recognized to be driven by lactate. As would be expected, lactate production in our studies was associated with progressive acidification of the medium. We used the chemical shift of histidine signals,^13^ a component of RPMI medium, to determine the *in situ* changes in pH during each acquisition (Supplementary Figures S4A and B). As shown in Supplementary Figure S4C, pH decreased from ~7.8 to 6.5 over a 24-h time course and inversely correlated with accumulation of lactate. NMR experiments with CLL cells in bicarbonate buffered RPMI agarose medium supplemented with 25 mM HEPES buffer demonstrated that lactate production was unaffected by the stabilization of extracellular pH (Supplementary Figure S4C).

### Primary CLL cells adapt to and tolerate extreme hypoxia

**Figure 2:**
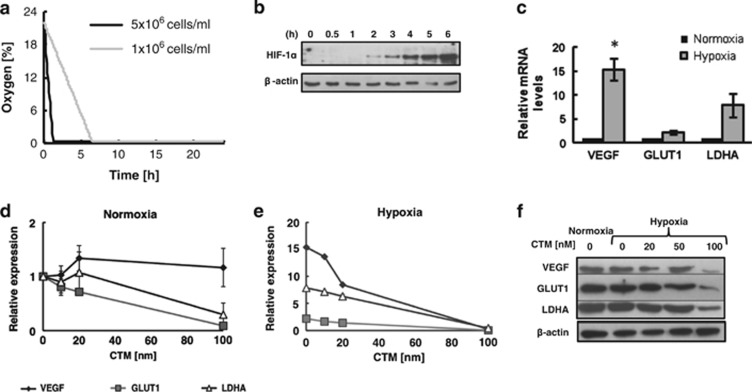
Level of HIF-1α increases in hypoxia together with the expression of its target genes that can be blocked by CTM. (**a**) Oxygen levels in NMR tubes were measured every 10 min using an oxygen probe placed inside the NMR tube throughout time-course experiments. Data are shown for one CLL sample at cell densities of 1 × 10^7^ and 5 × 10^7^ cells per ml and is representative of *N*>6 samples. (**b**) HIF-1α protein levels were determined at different time points. Cells were incubated in agarose matrix in an NMR tube at 37 °C and Laemmli buffer added directly to the tube at different timepoints to lyse cells without exposing them to oxygen. Western blot was performed using the anti-HIF-1α antibodies. Representative data from *N*=4 CLL samples. (**c**) QRT-PCR analysis of VEGF, GLUT1 and LDHA expression in CLL cells incubated in normoxia or hypoxia (in the NMR tube) for 24 h. Values are normalized to the normoxia control =1. Data are mean±s.e.m. of *N*=5 CLL samples; *P<0.05 by unpaired t student's test. QRT-PCR analysis of VEGF, GLUT1 and LDHA expression in CLL cells pre-treated for 3 h with increasing doses of CTM before incubating for 24 h in (**d**) normoxia or hypoxia (**e**). Values are normalized to the normoxia control without CTM. Data are mean±s.e.m. of *N*=5 CLL samples. (**f**) CLL cells were either incubated in normoxia, or pre-treated with a dose-titration of CTM for 3 h and then incubated for 21 h in hypoxia, before western blot analysis of VEGF, GLUT1 and LDHA protein levels. Data shown are representative of *N*=3 CLL samples.

Oxygen levels were measured in the NMR tube over the 24 h period using oxygen electrodes. As can be seen in Figure 2a, there was a cell number dependent decrease in oxygen levels with [O_2_] dropping below 3% within 1.5 h and ~6 h when 5 × 10^7^ per ml cells or 1 × 10^7^ per ml cells were used, respectively. The oxygen consumption rate appeared linear whilst oxygen was available. Consistent with the transition to hypoxia, western blot analysis showed detectable HIF-1α protein levels in CLL cells between 1 and 2 h in the NMR tube (Figure 2b) and levels continued to increase over the next 5 h. QRT-PCR analysis showed that mRNA expression of HIF-1α target genes (LDHA, VEGF and GLUT1) was detectable in CLL cells both in oxygenated and hypoxic conditions and that the expression increased in hypoxia (Figure 2c). The mRNA levels of GLUT1 and LDHA in oxygenated conditions were sensitive to the HIF-1α inhibitor CTM whereas expression of VEGF was not (Figure 2d). However, the elevated expression of all three mRNAs in hypoxia was sensitive to inhibition by CTM (Figure 2e). These data were corroborated at the protein level by detection of high levels of LDHA, VEGF and LDHA protein in primary CLL cells even under oxygenated conditions and reduction in protein levels for all three HIF-1α targets in the presence of increasing amounts of CTM (Figure 2f). This is consistent with previous reports that HIF-1α is active in circulating CLL cells despite the normoxic environment of peripheral blood.^14^ However, they also demonstrate that the HIF-1α axis is rapidly and sensitively elevated upon transition to hypoxia. Immunofluorescence and immunohistochemical staining of primary CLL cells confirmed low levels of HIF-1α in normoxic CLL cells with significantly elevated HIF-1α nuclear staining in hypoxia (Supplementary Figures S5 and S6).

### Primary CLL cells exhibit reversible metabolic plasticity during the transition between different oxygen environments

**Figure 3:**
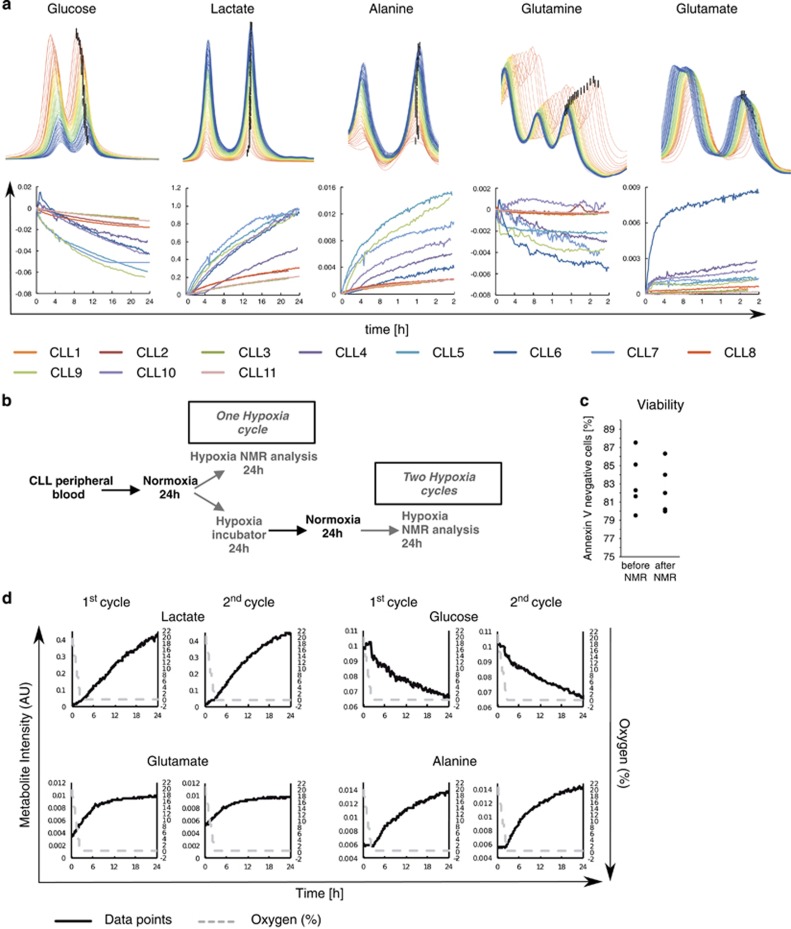
Real-time changes in metabolite peak intensities during 24 h primary CLL NMR time-course. (**a**) Representative superimposed fragments of spectra of a 24-h time course is shown for some metabolites. The first spectra were colored in red and the last spectra in blue. The black bar indicates the location of each peak that was used for kinetic analysis. Graphs show the intensity difference between the first and the following peaks from the spectra acquired over 24 h for 11 primary CLL samples. (Additional metabolites are shown in Supplementary Figure S7). (**b**) Schema of an experiment that demonstrates metabolic plasticity of CLL cells. Primary-CLL mononuclear cells were isolated from peripheral blood and incubated for 24 h in normoxia. Then the sample was split into two, one-half was analyzed in the NMR for 24 h (hypoxia) (first cycle) and the other half of the sample was incubated for 24 h in a hypoxic incubator, then for another 24 h in normoxia and finally analyzed in the NMR (hypoxia) for a further 24 h (2nd cycle). (**c**) Viability data for five primary CLL samples following completion of NMR after having undergone either one or two hypoxic cycles. (**d**) Representative NMR time-course data for one CLL sample of *N*=6. Intensity change for lactate, glucose, glutamine and alanine are shown for the cells during the first and the second hypoxic cycle. The dashed line represents oxygen concentration in the NMR tube during the experiment. (Additional metabolites shown in Supplementary Figure S8. Kinetic values corresponding to the time course are shown in Supplementary Table S2).

Real-time measurements over 24 h identified that levels of certain metabolites changed over time, for example, glucose, alanine, lactate, glutamine and 3-hydroxybutyrate (Figure 3a and Supplementary Figure S7). Glutamate variably accumulated across all the samples with one sample displaying particularly marked accumulation (Figure 3a). Other metabolites, including lysine, arginine and tyrosine, were remarkably stable during the acquisition of spectra (Supplementary Figure S7). The production of lactate was inversely correlated with glucose consumption (Figure 3a) with some CLL samples consuming glucose more rapidly than others. Concordantly, the production of lactate was reciprocally greatest in those samples that consumed most glucose.

Interestingly, of the six CLL samples with the slowest glucose consumption, five were clinical Binet stage A0, the least advanced CLL stage whereas there was only one A0 sample in the mid-glucose turnover group (Supplementary Table S1). Furthermore, all A0 CLL samples clustered in the low glutamine consumption group. Production of alanine also mirrored the extent of glucose consumption and lactate production. Similarly the consumption of glutamine mirrored glucose consumption and lactate production, suggesting that tricarboxylic acid (TCA) cycle activity depends at least in part on anaplerotic glutamine catabolism. Although sample numbers were too small for a general conclusion, these observations appear to indicate that CLL may be divided into tumors with higher and lower metabolic states that reflect disease stage (Figure 3a).

**Figure 4:**
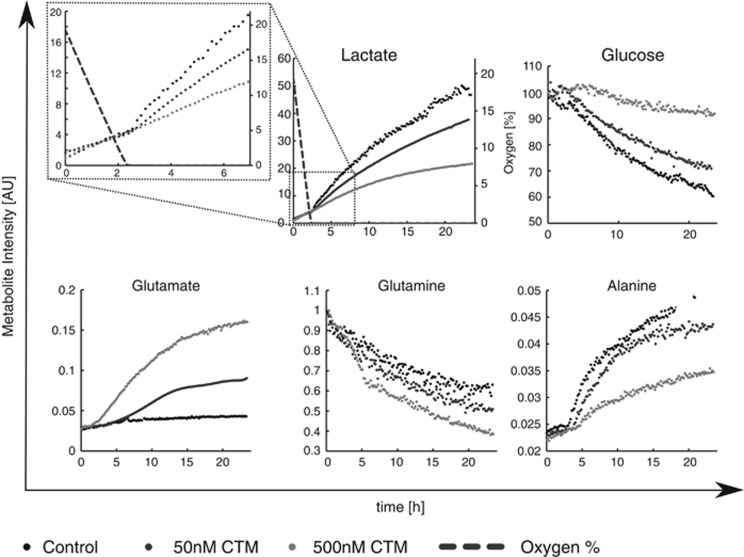
Metabolic adaptation of CLL cells to hypoxia involves HIF-1α. Representative NMR time-course data for a CLL pre-treated for 3 h with either 0, 20 or 100 nM CTM, before transferring into NMR for a further 24 h. Dashed lines on the lactate graph show oxygen levels inside the NMR tube. The top left panel shows an expanded view of lactate kinetics during the first 6 h with a visible shift after oxygen depletion which is inhibited by CTM. Data shown are representative of a minimum of *N*=3 CLL samples.

Key metabolites displayed discontinuity in their kinetics as oxygen became depleted (Figures 3d and 4). This discontinuity is indicative of a rapid adaptation of metabolism to lower oxygen levels with response times of minutes or less. These rate changes included increased accumulation of lactate and increased extracellular acidification rate with a transition to elevated anaerobic glycolysis indicating that CLL cells display depending on oxygen availability, an adaptive Warburg effect. There was also a marked onset of alanine synthesis upon entry into hypoxia (Figures 3 and 4). These rate changes appeared to be tightly associated with the transition to hypoxia.

The above observations indicate that CLL cells adapt to an environment of depleted oxygen using coordinated changes in metabolism and activation of HIF-1α. It is however important to note that lactate production was observed before entry into hypoxia, an observation that is again consistent with basal HIF-1α in oxygenated CLL cells. The kinetics in the increase of lactate production post entry into hypoxia appeared more rapid than the increases in HIF-1α protein levels but this may relate to differences in the sensitivity of the different measurements.

*In vivo,* CLL cells circulate between hypoxic (lymph nodes, spleen and bone marrow) and normoxic (blood) tissue compartments. Thus, if the changes observed in our experiments are physiologically relevant it would be expected that they are also reversible. To test this we performed real time NMR on cells from the same CLL sample that had either been: (1) transferred into an NMR tube for 24 h after an initial period at normoxia (cycle 1) or (2) after incubation in oxygenated conditions, transferred to a hypoxia incubator for 24 h then returned to oxygenated conditions for 24 h before finally placing them in the NMR tube for analysis of a second transition into hypoxia (cycle 2) (Figure 3b). Remarkably, viability of primary CLL cells was unaffected by transition between oxygen states (Figure 3c). As shown in Figure 3d and S8 (Supplementary Table S2), the kinetics of glucose and glutamine consumption, as well as lactate, glutamate and alanine production during between cycle 1 and cycle 2 treated cells were comparable. These observations indicate that CLL cells can repeatedly adapt to an environment of depleted oxygen using coordinated changes in metabolism.

### HIF-1α inhibition reverses changes in metabolism associated with hypoxia

To clarify whether this adaptation is HIF-1α dependent we used the HIF-1α inhibitor CTM. Consumption of glutamine was enhanced by CTM whereas consumption of glucose was attenuated by HIF-1α inhibition (Figure 4). We attempted to determine whether CTM caused preferential killing of CLL cells in hypoxia. However, exposure of CLL cells to CTM for 48–72 h induced cell death in the presence or absence of hypoxia (Supplementary Figure S9). This may relate to the aforementioned activity of HIF-1α in CLL cells in both normoxia and hypoxia.^14^ Furthermore, CTM inhibits the formation of functional HIF-1α/HIF-1β/p300(CBP) transcriptional complexes by acting upon the p300 coactivator.^15^ The actions of p300 as a coactivator are not restricted to HIF signaling and other p300 signaling pathways in CLL include the NFκB pathway.^16^ Therefore, the CLL cell death associated with CTM treatment may also be attributed to this or some other non-HIF function of p300 that is invariant between normoxia and hypoxia.

### Pyruvate is a key metabolite in the transition of CLL cells to hypoxia

**Figure 5:**
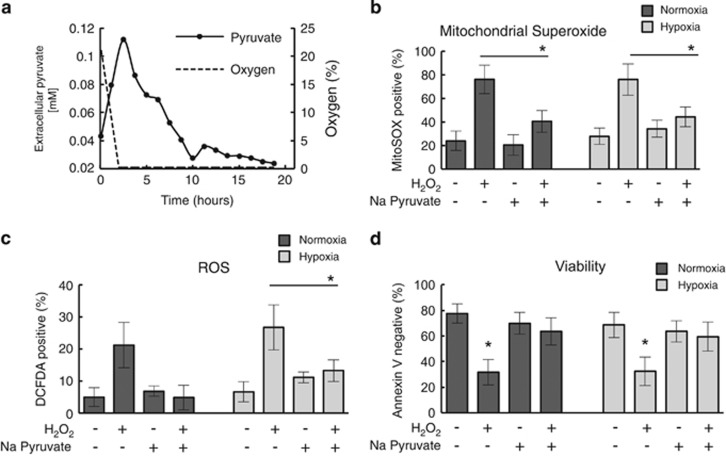
Pyruvate reduces mitochondrial superoxide and reactive oxygen species (ROS) level in CLL cells. (**a**) Real-time NMR analysis of pyruvate in CLL cells transitioning into hypoxia. Representative plot of extracellular pyruvate concentration together with the oxygen decrease during a CLL real time live cell NMR time course experiment. Data shown are mean of *N*=6 CLL samples. (**b**–**d**) CLL cells were incubated for 24 h with/without 10 mM H_2_O_2_ and/or 5 mM sodium pyruvate in normoxia or hypoxia (0.1% O_2_) before staining with (**b**) MitoSOX-Red for detection of mitochondrial superoxide; (**c**) DCFDA for detection of other ROS, or (**d**) Annexin V/PI to determine cell viability. Data are the mean±s.e.m. from *N*=5 CLL samples; *P<0.05 by unpaired Student's *t-*test.

Lactate continued to accumulate over the 24 h that we recorded spectra and reciprocally glucose was continually consumed. Similarly, once initiated in hypoxia, alanine accumulation continued throughout the experiment. In stark contrast, pyruvate kinetics were more complex. During the early stages, before oxygen depletion, pyruvate signals were seen to increase and subsequently to fall again during the period in hypoxia (Figure 5a), suggesting a key differential functional importance of this metabolite in oxygenated and hypoxic conditions. These observations were made after correcting the pyruvate peak intensity for an underlying glutamate resonance by calculating the glutamate peak intensity from other glutamate peaks in the spectra (Supplementary Figure S10).

Interestingly, exposure of CLL cells to CTM indicated the transition in pyruvate dynamics was largely independent of HIF activation (Supplementary Figure S11). NMR metabolic footprint analysis of media taken from CLL cells cultured in either oxygenated conditions or hypoxia demonstrated that CLL cells release pyruvate in the presence of oxygen but not in hypoxia indicating that the fall in pyruvate in hypoxia relates to reuptake of pyruvate into CLL cells (data not shown). Consistent with this, incubation of CLL cells with ^13^C-pyruvate in hypoxia demonstrated pyruvate uptake by CLL cells with transfer of ^13^C label to both lactate and alanine (data not shown).

Pyruvate has been demonstrated by others to directly protect cells against hypoxic stress.^17, 18^ We therefore hypothesized that CLL cells utilize pyruvate in hypoxia as a form of defense against hypoxia induced oxidative stress. To test the ability of CLL cells to utilize exogenous pyruvate for protection against oxidative stress we treated CLL cells with H_2_O_2_ inducing oxidative stress in the presence and absence of exogenously added pyruvate. As shown in Figures 5b and c and Supplementary Figure S12, exposure of CLL cells to 10 mM H_2_O_2_ resulted in elevated reactive oxygen species, including mitochondrial superoxide, both in hypoxia and normoxia. However, supplementation of the media with exogenous sodium pyruvate significantly diminished reactive oxygen species levels back to those observed in untreated cells. This effect is independent of whether cells are under normoxic or hypoxic conditions. Likewise, provision of exogenous pyruvate reversed H_2_O_2_-induced CLL cell killing (Figure 5d). This observation supports the view that pyruvate reduces oxidative stress and that this causes the time course of pyruvate levels observed in our experiments. It would also indicate that it is the generation of oxidative stress that is, the driver of pyruvate reuptake rather than the transition to hypoxia *per se*.

## Discussion

An increasing number of studies have shed light on specific characteristics of cancer metabolism. However, nothing is known about the kinetic changes involved in these metabolic adaptations and in particular the adoptive adaptations associated with changing microenvironments. This study shows that NMR is uniquely capable of monitoring such changes in real time using primary patient cells. Such experiments open new avenues for studying drug responses using primary patient cells.

These studies are important in CLL for which there remains no cure. However, CLL represents an excellent generic model of wider human B-cell malignancies. Their accessibility provides a unique opportunity to study primary cancer cells. Many agents that are effective in CLL are also effective in B-cell lymphomas, for example the CD20 targeting antibody rituximab.^19^ Therefore, studies in CLL are likely to inform the development of therapies in settings beyond this disease. Effective inhibition of metabolic processes is likely to add to current treatment opportunities. However, we would argue that CLL cells provide a wider model of human cancer and in particular a model in which to understand the processes of metastasis.

Although circulating CLL cells are arrested in G0/G1, most of these cells have undergone previous cell divisions within malignant lymph nodes.^20^ This indicates that CLL cells can oscillate between being ‘in' and ‘out' of cell cycle. Cells out of cell cycle are often described as ‘quiescent'. However, in the age of metabolomics, the definition of quiescence is likely to change. In terms of cell cycle, ‘quiescence' relates to being in a cell cycle state termed G_0._ Curiously, cells that are in G_0_ are not necessarily metabolically quiescent. Evidence that cell cycle quiescence may not be associated with metabolic quiescence has also been shown in ‘out of cycle' fibroblasts that maintain comparable metabolic rates to the proliferating cells.^21^

Our experiments demonstrate that primary CLL cells, which are non-cycling also maintain a high level of metabolic activity involving glycolysis and TCA cycle activity with associated O_2_ consumption and TCA cycle activity. Moreover, CLL cells reveal an unexpected metabolic plasticity even when in G0/G1, specifically a reversibly adaptive Warburg response where glucose consumption and lactate production is present in oxygenated conditions but rates change reversibly upon transition to hypoxia (Supplementary Figure S12).

Although oxygen was available, the TCA cycle appeared to be supported by glutaminolysis as evidenced by consumption of glutamine and O_2_, associated with the production of glutamate, pyruvate, lactate and alanine. It is interesting to note that HIF-1α inhibition using CTM impacted upon CLL cells in hypoxia, accelerating glutamine consumption and glutamate production, whereas diminishing glucose consumption and lactate production. These findings suggest that hypoxia induced HIF-1α activity acts to sustain glycolysis, as CLL cells transit from oxygenated to hypoxic environments and that lactate production is largely mediated by the consumption of glucose.

Importantly, real-time NMR time-courses of glucose, lactate, alanine and glutamine (Figure 3) revealed potential metabolic subtypes among the CLLs. In A0 CLL samples, the least aggressive subtype of CLL associated with lymphocytosis but no lymphadenopathy, glucose consumption was low, glutamine consumption was equally low and lactate/alanine production showed equally low metabolic activity. For one patient sample we observed exceedingly high glutamate production. Although not the aim of this study, these data suggest that metabolic subtypes exist which may correlate with clinical phenotype and may provide information regarding biomarkers. However, patient numbers are still too small in this study to demonstrate this unequivocally.

We also observed HIF-1α independent differential utilization of pyruvate in oxygenated and hypoxic conditions. When oxygenated, CLL cells exported pyruvate. However, as oxygen concentration dropped below 1%, CLL cells imported pyruvate. Our data would indicate that this pyruvate import is in response to hypoxia-associated oxidative stress rather than hypoxia *per se*, as CLL cells imported pyruvate when treated with H_2_O_2_ under normoxic conditions. In the environs of the CLL lymph node, the tumor cells may not be the only source of pyruvate.

Our study is one of a growing number of studies that have analyzed metabolism in living cells^22, 23^ and to our knowledge the first to study real-time metabolism in primary patient cancer cells. Using these cells, we observed fast metabolic adaptation to niche conditions using a simple model of oxygen depletion. By embedding cells in a dilute agarose matrix in an NMR tube we restricted oxygen access while preserving cells in a non-proliferating viable state. The agarose matrix prevents sedimentation of cells thus preserving homogeneity of the NMR sample, an important prerequisite to obtain high-resolution NMR spectra. A low density agarose matrix does not affect the mobility of small molecules in any significant way thus preserving small line widths. Using this experimental arrangement a time resolution of 5–10 min is feasible to observe extracellular metabolites using a 600 MHz spectrometer, sufficient to see metabolism arising from 5–10 × 10^6^ primary blood cancer cells.

However, equally important has been the integration in our study of NMR methodology with cancer biology, revealing previously unknown plasticity of primary CLL cell metabolism, an observation that is likely to open new therapeutic avenues in CLL cells. Moreover the properties of CLL cells that permit metabolic plasticity in different microenvironments may be recapitulated in metastatic cells of other cancers. Beyond this, we have identified potential evidence that CLL cells basal metabolism displays heterogeneity from patient to patient; an observation that would not be possible using established cell lines. A future larger scale study would permit correlation of metabolic activity with disease parameters such as disease stage, prior or ongoing treatment, as well as correlative studies with disease progression and association with prognostic markers.

Finally, the methodology presented here has considerable potential for applications in personalized medicine. Unlike most other analytical technologies, NMR is completely non-invasive and preserves cells. This opens new avenues to test the effect of treatment options ‘*ex vivo*' using primary patient cells, which can afterwards be further characterized.
