# Supplementary material for: Metabolic plasticity in CLL: adaptation to the hypoxic niche
Source: Leukemia. 2015 Sep 4;30(1):65–73. doi: 10.1038/leu.2015.187 (PMC4705428; doi:10.1038/leu.2015.187)
Supplement: Supplementary Information [file leu2015187x1.pdf]

Supporting Information

Metabolic plasticity in CLL: Adaptation to the hypoxic niche

Katarzyna M. Koczula[a], Christian Ludwig[a], Rachel Hayden[b], Laura Cronin[b], Guy Pratt[a,d], Helen Parry[a], Daniel Tennant[a], Mark Drayson[c], Christopher M. Bunce[b], Farhat L. Khanim[b*], Ulrich L. Günther[a,1*]*

[a]School of Cancer Sciences, [b]School of Biosciences, [c]School of Immunity and Infection, University of Birmingham, Birmingham B15 2TT, United Kingdom, [d]Department of Haematology, Birmingham Heartlands Hospital, Birmingham, United Kingdom.

[*]made equal contributions
[1]To whom correspondence should be addressed:
u.l.gunther@bham.ac.uk

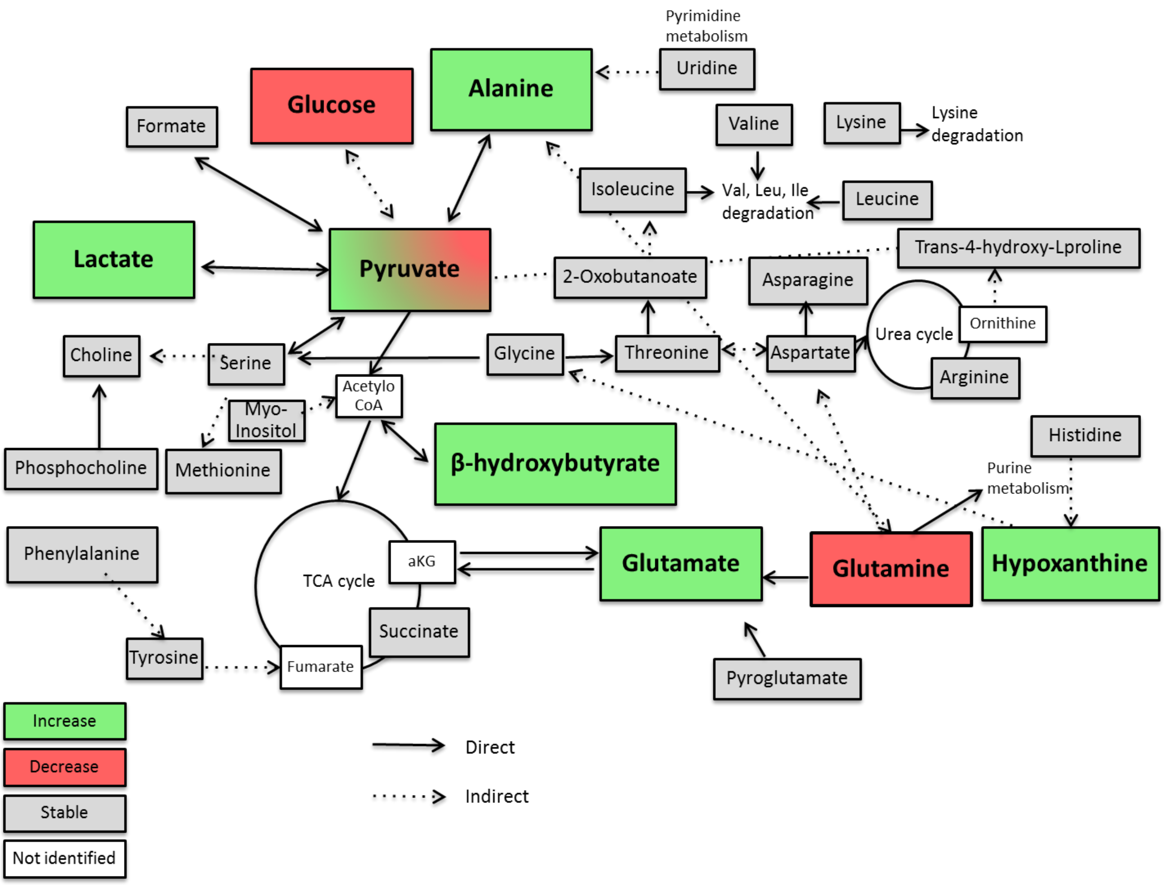

Figure S1. Metabolic map presenting all the metabolites assigned in the ^{1}H NOESY spectra recorded on samples with primary CLL cells. Most of the metabolites identified in the spectra and presented on this map are part of the formulation of RPMI medium with the exception of : lactate, pyruvate, β-hydroxybutyrate, hypoxanthine, pyroglutamate, formate, succinate, uridine, 2-oxobutanoate, choline, phosphocholine, Trans-4-hydroxy-L-proline and uridine.

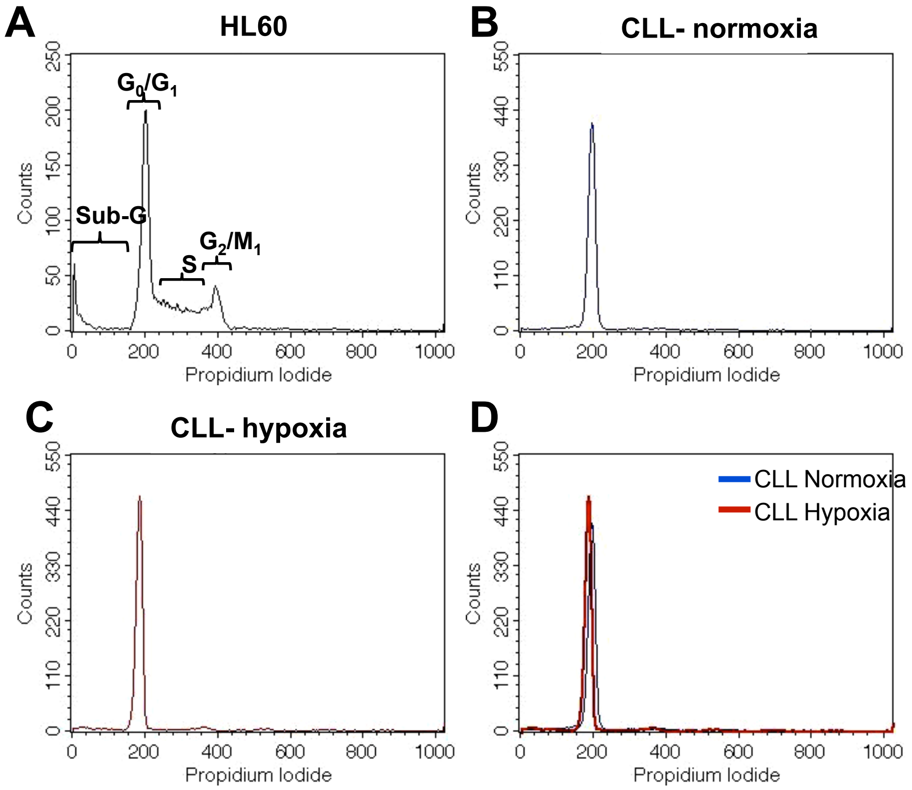

Figure S2. **CLL cells remains in G_0/G_1 phase of cell cycle in both normoxia and hypoxia.**

Cell cycle analysis of 10,000 CLL cells assessed by propidium iodide staining of cells followed by flow cytometry analysis. A) Example of proliferating cells, HL60 acute myelogenous leukaemic cell line, with cells in different cell cycle stages (as marked). CLL cells in B) normoxia and C) hypoxia are in $G_{0/}$ G_1 cell-cycle phase. D) Superimposed data from CLL cells in normoxia and hypoxia.

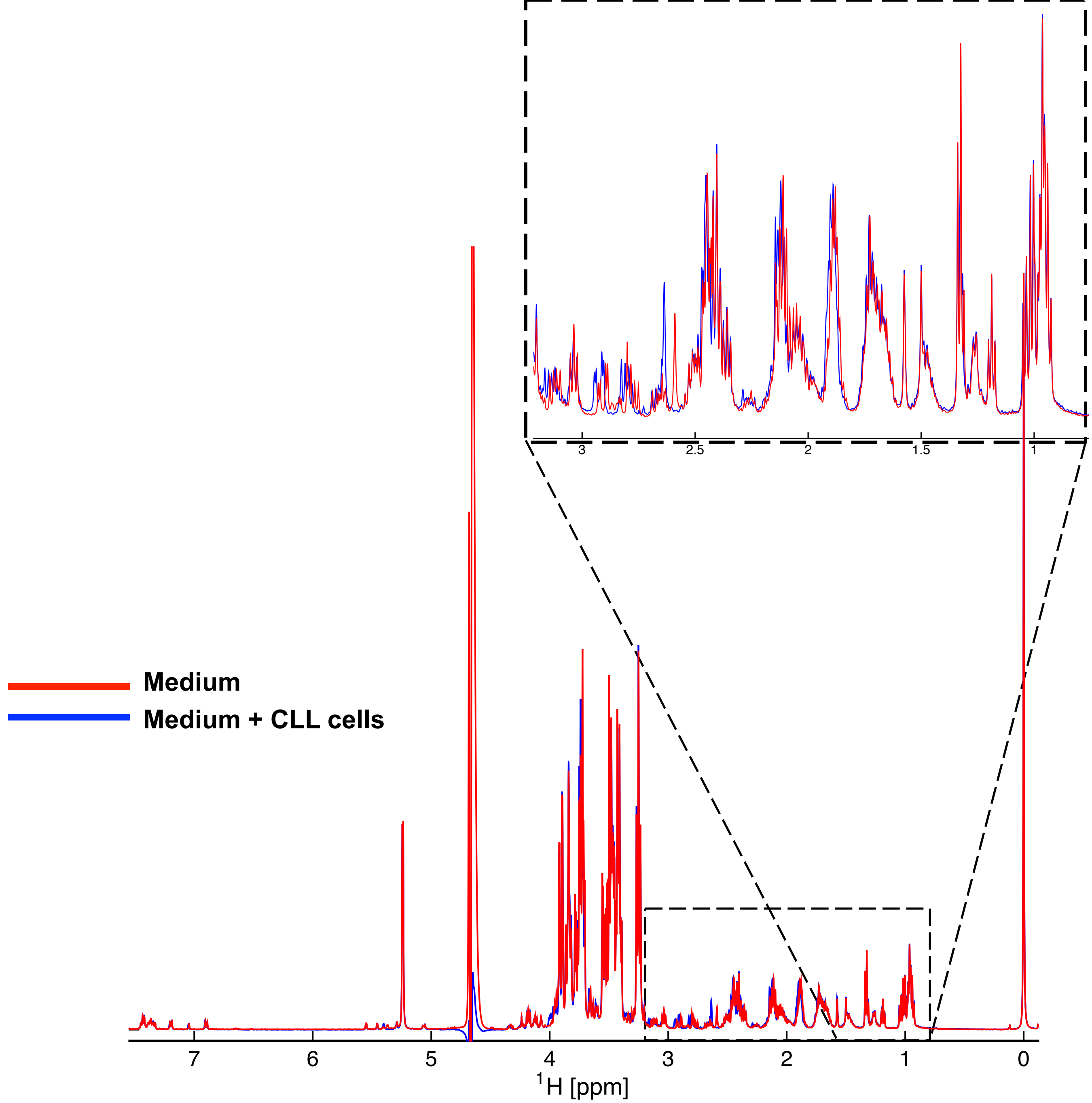

Figure S3. Spectrum of CLL cells in medium with agarose overlayed with spectrum of medium alone.

The blue spectrum is the last spectrum collected from 1x10^7 CLL cells embedded in RPMI medium supplemented with 1% ITS+ and 0.1% low melting agarose after a 24h NMR experiment. After acquiring the spectrum, the sample was centrifuged to pellet agarose and cells. Medium supernatant was transferred back to an NMR tube and spectrum collected (red). Fragment from 0.5- 3.5 ppm is enlarged and presented in the top rectangle.

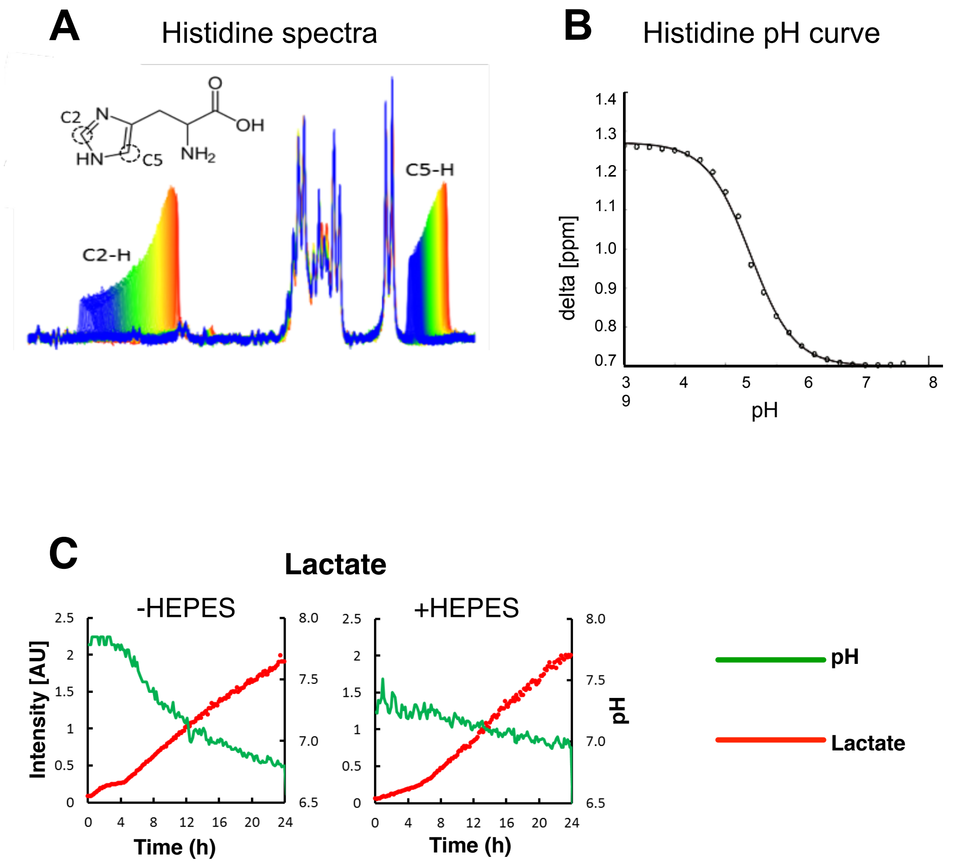

Figure S4. Determination of pH in NMR tube using histidine peaks.

A) Part of the spectrum containing two histidine peaks corresponding to protons connected to carbons C2 and C5 of histidine. Spectra collected over 24h are superimposed and coloured with the first spectra coloured in red and the last spectra in blue. The difference in chemical shift observed for these two peaks corresponds to pH in the sample.

B) A histidine pH curve was calculated by plotting the difference in chemical shift of the two histidine peaks (C2-H vs C5-H) in RPMI media which had been adjusted to a pH range covering pH3-9. Data shown is mean data from N=3 pH experiments. The pH value was calculated according to $pH=5.49+\log10((\delta-1.272)/(0.7004-\delta))$ for each time point of the NMR time-course experiments.

C) Representative plots of pH overlaid with lactate intensity over 24h for a sample incubated in standard RPMI medium either with or without 25mM HEPES.

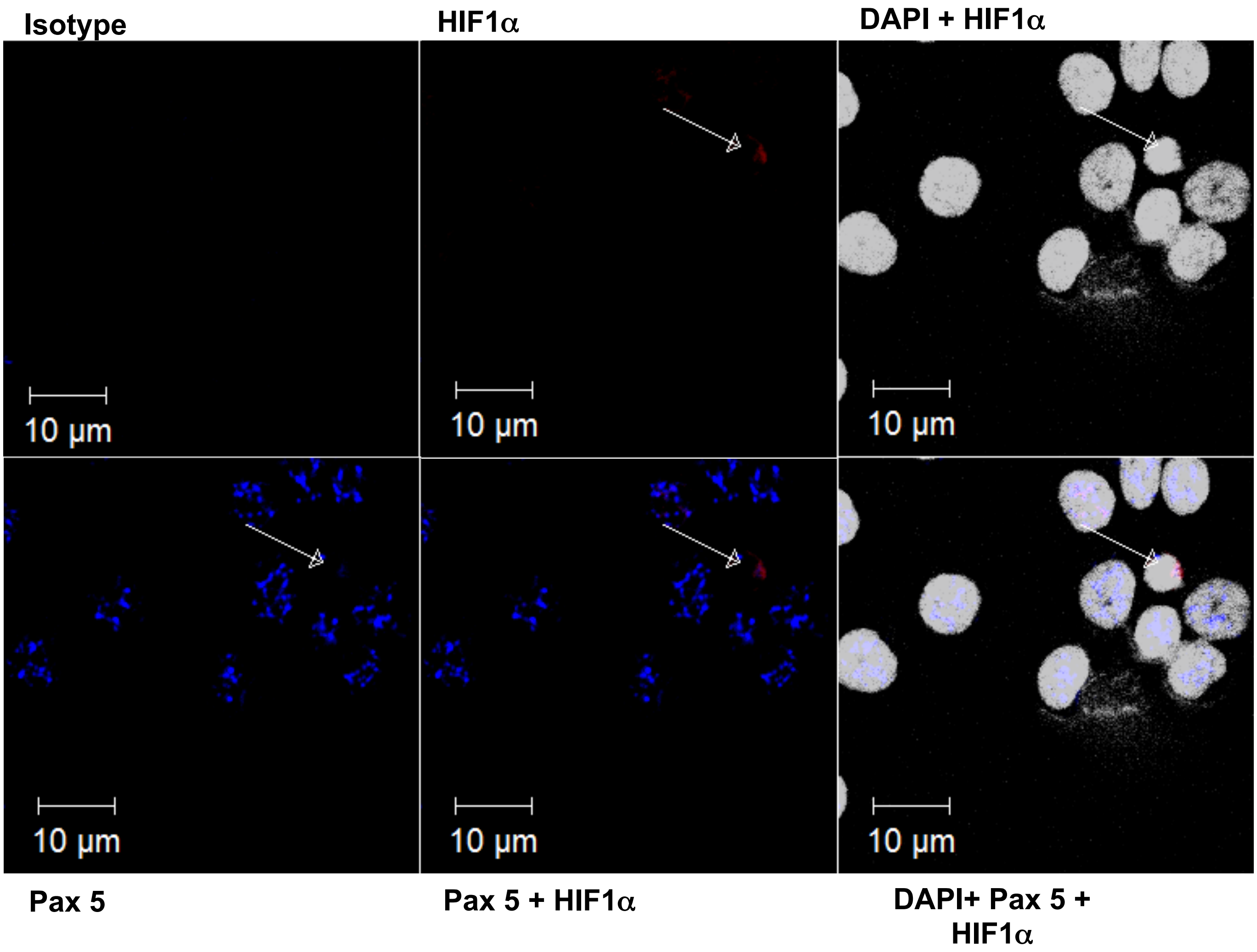

Figure S5. HIF-1α is present at low levels in the cytoplasm of normoxic CLL cells. Cytospins of primary CLL cells pre-incubated in normoxia for 6h were fixed with acetone and then stained with anti- HIF-1α antibody (red)(Sigma), anti-PAX-5 (blue)(R&D systems) to identify CLL cells and DAPI nuclear counterstain (white). Image shown is representative of N=3 CLLs.

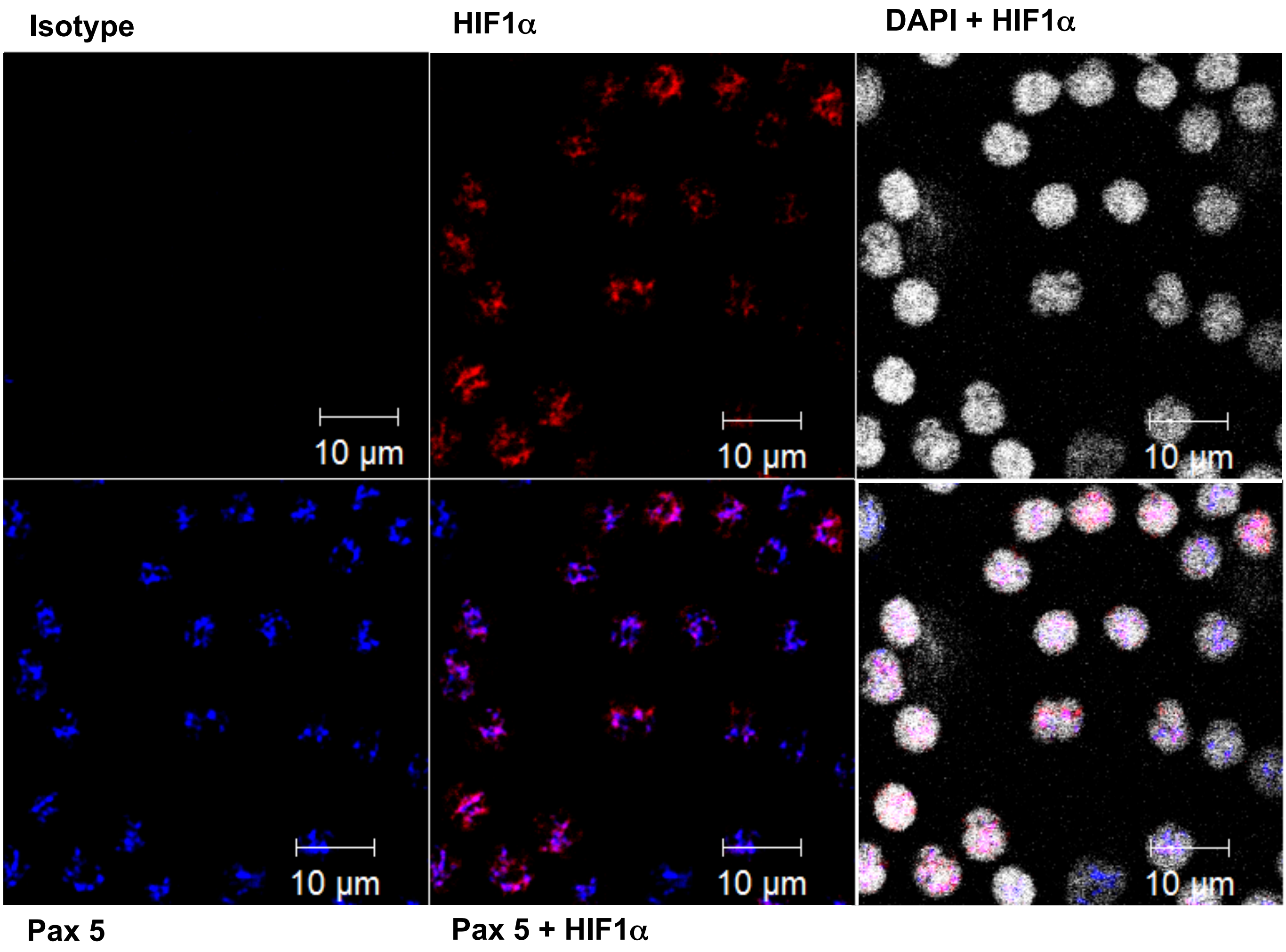

Figure S6. HIF-1α is present at low levels in the nuclei of hypoxic CLL cells.
Cytospins of primary CLL cells pre-incubated in 0.1% O_2 for 6h were fixed with acetone and then stained with anti- HIF-1α antibody (red) (Sigma), anti-PAX-5 (blue) (R&D systems) to identify CLL cells and DAPI nuclear counterstain (white). Image shown is representative of N=3 CLLs.

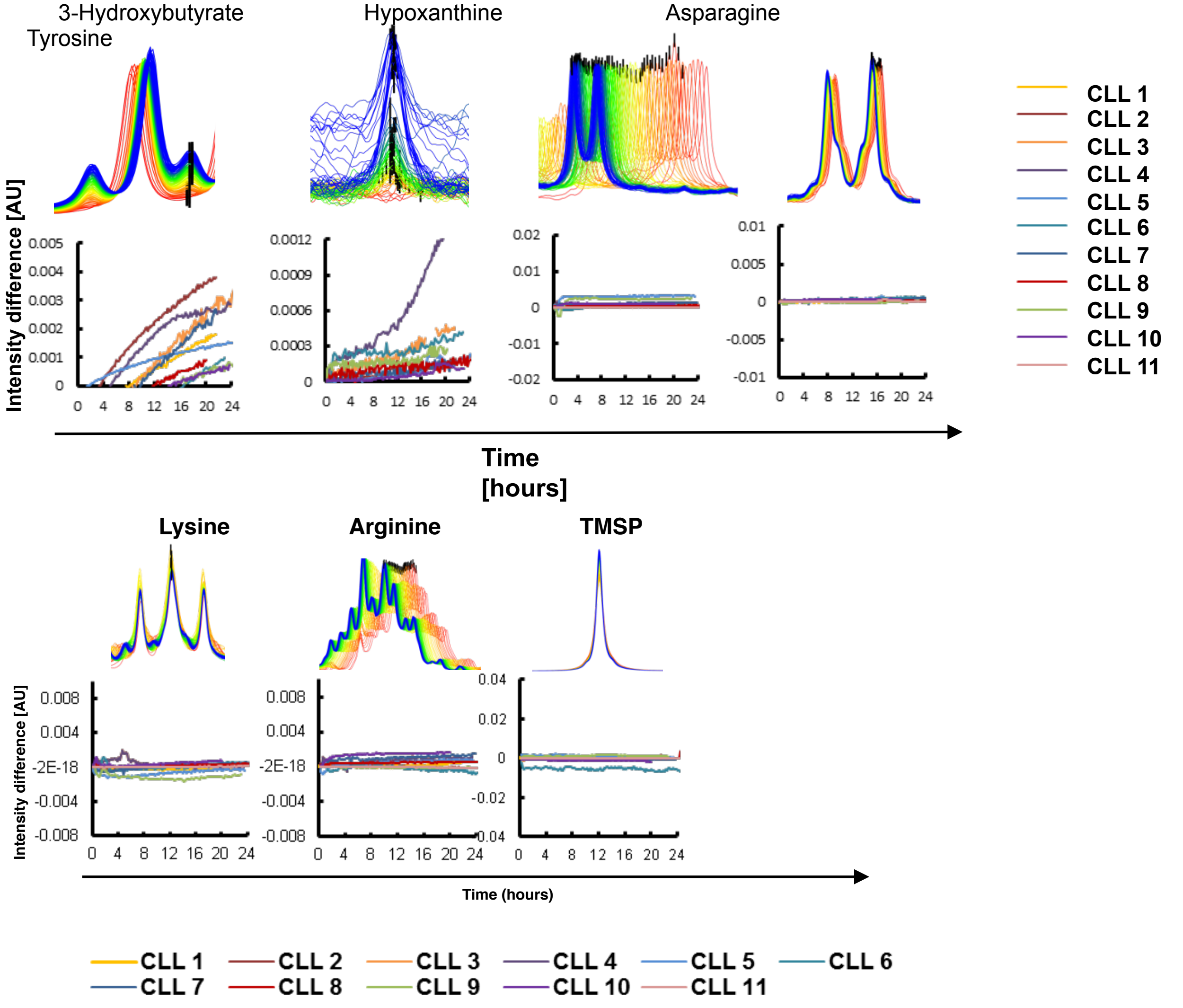

Figure S7. Real time changes in metabolite peak intensities during the NMR timecourse (additional metabolites, not shown in Figure 3).
Superimposed spectra with the peaks corresponding to the chosen metabolites. The spectra collected at the start of the experiment were coloured in red and the last spectra collected at 24h in blue. The black mark indicates the peaks that were used for kinetic analysis. The graphs show the intensity difference between the first and the following peaks from the spectra acquired during 24h for 11 primary CLL samples.

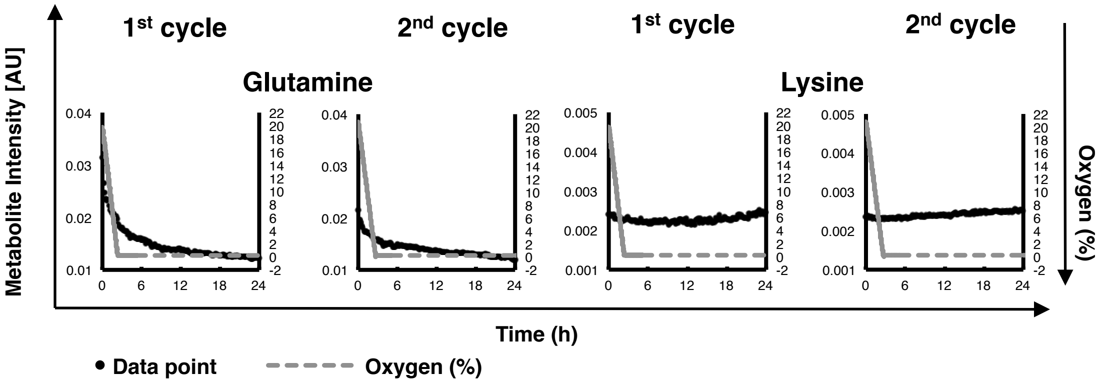

Figure S8. Real time changes in metabolite peak intensities during 24h primary CLL NMR time-course for additional metabolites (others shown in Figure 3). Additional NMR time-course data for one CLL sample. Intensity change for lactate, glucose, glutamine and alanine are shown for the cells during the first and the second hypoxic cycle. The dashed line represents the oxygen concentration in the NMR tube during the experiment.

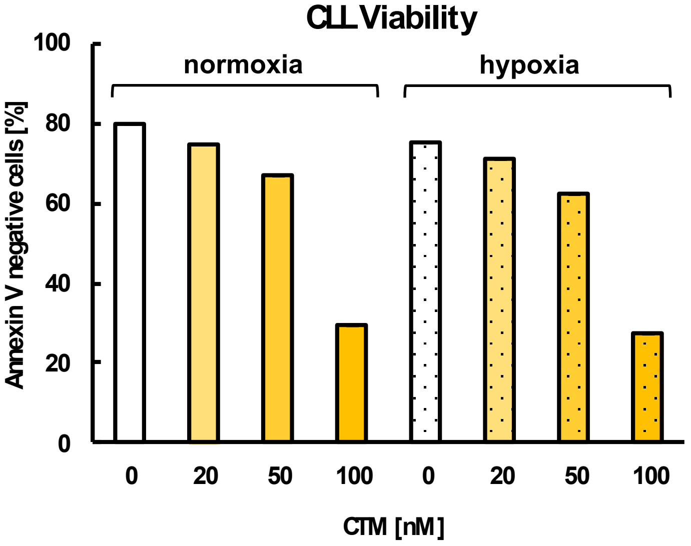

Figure S9. HIF-1α inhibition by chetomin (CTM) is toxic for CLL cells in both normoxia and hypoxia.

Viability of CLL cells after 48h incubation with CTM in normoxia or hypoxia. Cells were pre-treated with CTM for 3 hours before entering hypoxia for a further 21h. Data are mean of N=3 CLLs ± SEM.

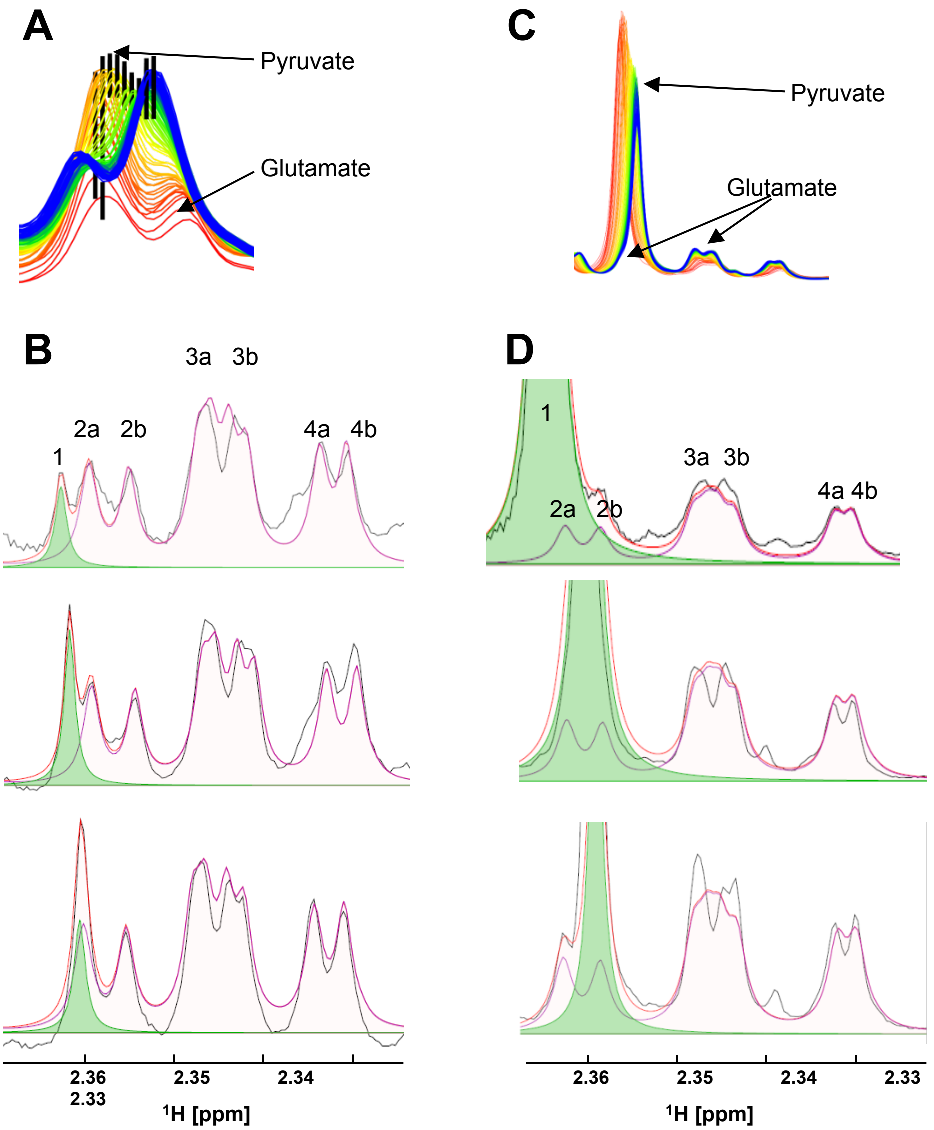

Figure S10. Analysis of pyruvate concentrations during real time NMR time-course experiments.

A) Superimposed time-course spectra with the pyruvate peak during the timecourse. As the pH decreases, the pyruvate peak shifts to the right and overlaps with one of the glutamate resonances. B) Using Chenomx software, we were able to assign pyruvate (green peak 1) and glutamate (purple peaks 2ab,3ab,4ab) peaks and estimate their concentrations. Glutamate concentration (corresponding to area under the curve) was estimated using glutamate peaks 2b, 3ab and 4ab. In order to estimate the pyruvate concentration, from the area under the curve of the overlapping pyruvate-glutamate peak, estimated area under the curve of peak 2b was subtracted. C) Sample with CLL cells was spiked with pyruvate, the same pH dependent shift is observed. D) Concentration of glutamate and pyruvate was estimated accordingly using Chenomx software.

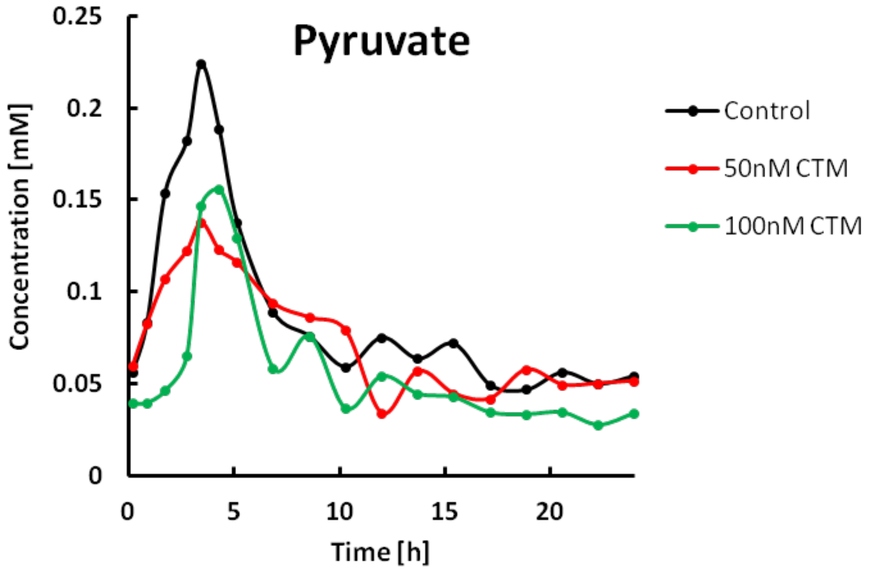

Figure S11. The transition in pyruvate dynamics was independent of HIF-1α activation

NMR time-course data for the control CLL experiment and cells treated with 20mM and 100nM chetomin (CTM). Cells were pre-treated with CTM for 3h before starting the NMR experiment. Graph presents changes of extracellular pyruvate concentration over time. Concentration values were calculated from the area under the curve using Chenomx software. Figure shows representative data from N=3 experiments.

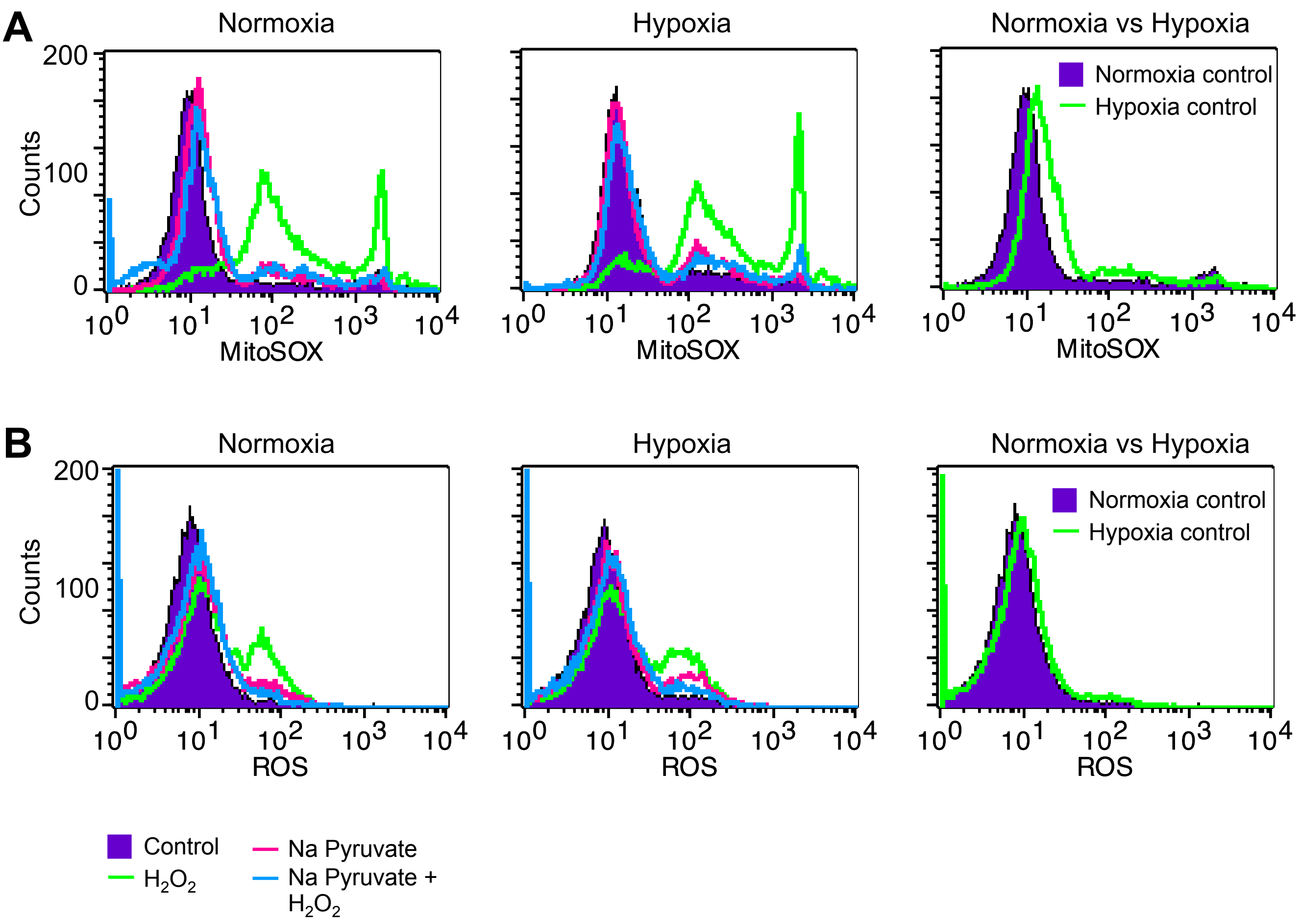

Figure S12. Exogenous pyruvate reduces mitochondrial superoxide and ROS level in CLL cells. CLL cells were incubated for 24h with 10mM H_2O_2 and 5mM sodium pyruvate in normoxia or hypoxia (0.1% O_2) prior to harvesting. Cells were transferred to FACS tube, washed and incubating with A) MitoSOX-Red for detection of mitochondrial superoxide or B) Carboxy H_2-DCFDA for detection of other ROS and analysed by flow cytometry. Figure shows representative histograms from N=3 experiments.

Patient	Sex	Age (Y)	Stage	Additional clinical features	Leukocyte count	CD19+ at the time of sampling [%]	Length of time with disease	Treatment when sampled	Previous treatment
1	M	85	C	CD38 neg, Zap70 pos, Normal cytogenetics.	150	91	14y 3m	Observation	First treated with chlorambucil in 1998 then in 2012.
2	M	71	A0	CD38 neg	150	90	2y 11m	Observation	none
3	M	58	A0	CD38 neg	58	74	1y 9m	Observation	None
4	F	92	C	ATM, p53 WT. CD38 unknown	191	88	18y 2m	Chlorambucil	24/9/02 then 04/07/08 and 2012
5	F	76	B	ATM and p53 WT -	170	85	6y 1m	Observation	None. progressive on that date though with sweats, LN and high WCC. No cytopenia
6	M	58	A	CD38 neg	58	80	1y 9m	Observation	None
7	F	77	A	nil	76	89	8y 9m	Observation	None. Had eyelid swelling but RT given locally after this date.
8	M	82	A0	nil	132	75	7y 9m	Observation	none
9	M	65	A	CD38 neg	147	59	2y 3m	Observation	none
10	F	92	A0	CD38 neg	60	76	18y 2m	Chlorambucil	none
11	M	83	A0	Additional 1q on karyotype. ATM, p53 WT	177	92.5	6y 11m	Observation	Previous FC in 2009 x 2 courses, BaP started week after this sample

Table S1. Summary of CLL patient clinical characteristics.
CLL samples used to obtain data presented in Figure 4 and S6. Patients were attending the outpatient clinic at Birmingham Heartlands Hospital or Queen Elizabeth Hospital in Birmingham, UK. Data shown is clinical information at the time that patient samples were used in this study.

Metabolite	First cycle				Second cycle			
	I_0	I_1	r	r_2	I_0	I_1	r	r_2
**Lactate (before hypoxia)**	0.079		0.046		0.062		0.051	
**Lactate (in hypoxia)**	3.971	4.325	0.0451		3.072	3.42	0.065	
**Glucose**	0.136	0.507	0.287	0.005	0.123	0.468	0.303	0.005
**Glutamine**	0.071	0.092	0.517	0.0009	0.0332	0.095	0.825	0.0009
**Alanine**	0.0758	0.058	0.088		0.07	0.058	0.118	
**Glutamate**	0.058	0.043	0.209		0.038	0.019	0.2	
**Pyruvate**	0.069	0.052	0.37		0.056	0.037	0.437	
**3-Hydroxy butyrate**	0.028	0.02	0.07		0.025	0.021	0.094	
**Hypoxanthine**	0.0004		0.038		0.0004		0.049	

Table S2. Kinetics of metabolite changes measured by real-time NMR during two alternate normoxia/hypoxia cycles.

Using the time series analysis user interface (TSATool) in the MetaboLab program, we applied the kinetic functions to fit data points to describe the changes in metabolite concentrations. For lactate, alanine, glutamate and 3-hydroxybutyrate we assumed a first order kinetics according to: $I(t)=I_0-I_1*e^{-rt}$ where I(t) is the current metabolite concentration at time point t, I_0 the theoretical metabolite concentration at infinite time, I_0 - I_1 the concentration at time point 0 and r the rate of change; for lactate before hypoxia I0*(1-exp(-r*t)) kinetics were applied as the concentration at timepoint 0 is close to 0; for glucose and glutamine the assumed kinetics was a bi-exponential decay to: $I(t)=I_0*e^{-rt}+I1-r_1t$ where I(t) is the current metabolite concentration at the (arbitrary) time point t, I_0 + I_1 is the starting concentration and r and r_1 are the rate constants describing the glucose usage. For hypoxanthine the x=a+r*t function was used approximating the linear part of a mono-exponential kinetics like the one assumed for lactate. r again is the rate constant. Those are kinetics fitted for the CLL data shown in Figure 6.
